# 

**Published:** 1845-07

**Authors:** 


### CONTENTS OF Nº XXXIX

OF THE

### British and Foreign Medical Review.

JULY 1845.

---

### PART FIRST.—Analytical and Critical Reviews.

PAGE

ART. I.—The Duality of the Mind proved by the Structure, Functions, and Diseases of the Brain, and by the phenomena of Mental Derangement, and shown to be essential to Moral Responsibility. By A. L. WIGAN, M.D. . 1

ART. II.—Traité de Pathologie cérébrale, ou des Maladies du Cerveau; nouvelles Recherches sur sa Structure, ses Fonctions, ses Altérations, et sur leur Traitement thérapeutique, moral et hygiènique. Par SCIPION PINEL, M.D. ancien médecin des aliénés de la Salpêtrière et de Bicêtre &c. . . 18

Treatise on Cerebral Pathology; containing original Researches on the Structure, Functions, and Morbid Changes of the Brain, and the therapeutical, moral, and hygienic Treatment of its Diseases. By SCIPION PINEL, M.D. formerly Physician to the Lunatic Department of the Salpêtrière and Bicêtre Hospitals, &c. &c.

ART. III.—A Treatise on Operative Surgery, comprising a Description of the various Processes of the Art, including all New Operations; exhibiting the state of Surgical Science in its present advanced condition. By JOS. PANCOAST, M.D., Professor of General and Descriptive Anatomy in Jefferson Medical College, Philadelphia . . . . . . . 39

ART. IV.—Urinary Deposits, their Diagnosis, Pathology, and Therapeutical Indications. By GOLDING BIRD, A.M. M.D. Assistant Physician to and Lecturer on Materia Medica at Guy's Hospital, &c. &c. . . . 58

ART. V.—Zur Analysis und Synthesis der pseudo-plastischen Prozesse im Allgemeinen und einiger im Besonderen. Von Dr. GUSTAV ZIMMERMANN 71

Contributions to the Analysis and Synthesis of Pseudo-plastic Processes. By Dr. G. ZIMMERMANN, Assistant-Surgeon in the Prussian Army.

§

4297

PAGE

ART. VI.—Medico-Chirurgical Transactions, published by the Royal Medical and Chirurgical Society of London. Vol. XXVII . . . 83

Mr. STANLEY on rupture of the ureter, &c. from external violence . ib.
Mr. OTTLEY on cysticercus cellulosæ of the brain . . . 84
Mr. DALRYMPLE on spermatozoa in hydrocele of the tunica vaginalis . ib.
Mr. HAWKINS on carcinoma of the thyroid gland . . . 85
Mr. B. COOPER on syncope from the admission of air into a vein . 87
Mr. WILSON on horn developed from the human skin . . ib.
Mr. DALRYMPLE on organization of coagula . . . 88
Mr. COOPER on extirpation of an ovarian cyst . . . 89
Mr. GREENHOW on removal of a diseased ovarium. . . 90
Dr. JONES on uric acid in the urine . . . . ib.
Dr. BURROWS on carcinoma of the lungs . . . ib.
Dr. WILSON on acute diseases of the throat and larynx . . 91
Dr. JONES on oxalate of lime in the urine . . . 92
Mr. PAGET on obstructions of the branches of the pulmonary artery . 93
Dr. DAVY on the composition of the meconium . . . ib.
Dr. H. ROE on paracentesis thoracis . . . . ib.
Dr. T. THOMSON's account of a case of empyema . . . ib.
Mr. HEWETT on omental sacs in strangulated herniæ . . 94
Dr. TODD on dissecting aneurism of the aorta, innominata, &c . ib.
Mr. HEY on aneurism of the external iliac . . . 96
Dr. REID on tubular expectoration from the bronchi in the adult . ib.
Dr. GREEN's tabular view of the seat of tubercle . . . 97
Mr. BARLOW on tumour in the right hypochondrium . . 99
Dr. WARREN on gelatiniform cancer . . . . 100
Mr. PAGET on a cyst containing seminal fluid . . . ib.
Dr. MERRIMAN's statistical records of the Asiatic cholera . . ib.
Mr. SHARP on necrosis of the lower jaw . . . ib.
Mr. SOLLY on the pathology of mollitis ossium . . . ib.
Mr. WORTHINGTON on fistula, simulating stone in the bladder . . 101
Mr. PHILLIPS's observations on extraction of ovarian tumours . ib.

ART. VII.—Causes générales des Maladies Chroniques, spécialement de la Phthisie Pulmonaire, et Moyens de prévenir la Développement de ces Affections, &c. Par A. FOURCAULT . . . . . 102

On the general Causes of Chronic Diseases, particularly Pulmonary Phthisis, and on the Means of preventing these Affections, &c. By A. FOURCAULT.

ART. VIII.—1. Handbuch der Chirurgie, zum Gebrauche bei seinen Vorlesungen. Von MAXIMILIAN JOSEPH CHELIUS . . . . 110

Manual of Surgery, for use at his Lectures. By M. J. CHELIUS.

2. A System of Surgery. By J. M. CHELIUS. Translated from the German, and accompanied with additional Notes and Observations, by JOHN F. SOUTH.

ART. IX.—The Actual Process of Nutrition and Inflammation in the Living Structure, demonstrated by the Microscope. Part II. By W. ADDISON, F.L.S. &c. 121

ART. X.—The General Nature and Treatment of Tumours. By GEO. MACILWAIN 127

ART. XI.—Traité d' Anatomie médico-chirurgicale et topographique, considérée spécialement dans ses applications à la Pathologie à la Médecine légale, à l'Obstetricie et à la Médecine opératoire. Par J. E. PETREQUIN . . 133

A Treatise on Medico-Chirurgical and Topographical Anatomy, considered specially in relation to Pathology, Medical Jurisprudence, Midwifery, and Operative Medicine. By J. E. PETREQUIN.

PAGE

ART. XII.—An Essay on the Philosophy of Medical Science. By ELISHA BARTLETT, M.D. . . . . . . . 140

ART. XIII —Beobachtungen über den Scorbut, vorzüglich in pathologisch-anatomischer Beziehung. Von G. v. SAMSON-HIMMELSTIERN . . 147

Observations on Scurvy, especially with reference to its Pathological Anatomy. By Dr. G. v. SAMSON-HIMMELSTIERN.

ART. XIV.—A Treatise on the Forces which produce the Organization of Plants. With an Appendix, containing several Memoirs on Capillary Attraction, Electricity, and the Chemical Action of Light. By JOHN WILLIAM DRAPER, M.D. . . . . . . 153

ART. XV.—A Physiological Essay on the Thymus Gland. By JOHN SIMON, F.R.S. 159

ART. XVI.—Thyrophymate Endemico per Nassoviam et Hessiam Electoralem. Dissertatio inaug. med. quam scripsit CAROLUS PHILIPPUS FALCK . 168

Thesis on the Endemic Bronchocele of Nassau and the electorate of Hesse. By C. P. FALCK.

ART. XVII.—Lectures on subjects connected with Clinical Medicine, comprising Diseases of the Heart. By P. M. LATHAM, M.D. . . . 175

ART. XVIII.—On the Diseases of the Jaws, with a brief Outline of their Anatomy, and a description of the Operations for their Extirpation and Amputation, with Cases and Illustrations. By R. O'SHAUGHNESSY, F.R.C.S.E. &c. . 195

ART. XIX.—A Collection of Cases of Apoplexy, with an explanatory Introduction. By EDWARD COPEMAN . . . . . 197

ART. XX.—Du Diagnostique Anatomique des Maladies du Foie et de sa valeur au point de vue thérapeutique. Thèse de Concours. Par MAXIME VERNOIS, M.D. 199

---

### PART SECOND.—Bibliographical Notices.

ART. I.—The Physiological Anatomy and Physiology of Man. By ROBERT BENTLEY TODD, M.D. F.R.S., and WILLIAM BOWMAN, F.R.S. . . . 201

ART. II.—Outlines of Military Surgery. By SIR GEORGE BALLINGALL, M.D. . 202

ART. III.—Practical Remarks on some exhausting Diseases, particularly those incident to Women. By SIR JAMES EYRE, M.D. . . . 203

ART. IV.—History of the York Dispensary, containing an Account of its Origin and Progress to the present time, comprising a period of fifty-seven years. By OSWALD ALLEN . . . . . . 204

ART. V.—Practical Observations on the Diseases most fatal to Children; with reference to the propriety of treating them as proceeding from Irritation and not from Inflammation. By P. HOOD . . . . 205

PAGE

ART. VI.—1. The Seven Books of Paulus Ægineta. Translated from the Greek. With a Commentary embracing a complete view of the knowledge possessed by the Greeks, Romans, and Arabians on all subjects connected with Medicine and Surgery. By FRANCIS ADAMS . . . 206

2. Observations on Aneurism, selected from the Works of the principal writers on that Disease from the earliest periods to the close of the last century. Translated and Edited by JOHN E. ERICHSEN, Lecturer on General Anatomy and Physiology at the Westminster Hospital . . . ib.

3. Animal Chemistry, with reference to the Physiology and Pathology of Man. By Dr. J. FRANZ SIMON, Fellow of the Society for the Advancement of Physiological Chemistry at Berlin, &c. &c. Translated and Edited by GEORGE E. DAY, M.A. . . . . . . ib.

ART. VII.—A Pentaglot Dictionary of the Terms employed in Anatomy. Physiology, Pathology, Practical Medicine, Surgery, Obstetrics, Medical Jurisprudence, Materia Medica, Pharmacy, Medical Zoology, Botany, Chemistry; in Two Parts: Part I—with the leading term in French, followed by the Synonymes in the Greek, Latin, German, and English; Explanations in English; and copious illustrations in the different Languages. Part II—A German-English-French Dictionary, comprehending the Scientific German Terms of the preceding Part. By SHIRLEY PALMER, M.D. . . . . 207

ART. VIII.—Introduction to the Study and Practice of Midwifery. By W. CAMPBELL, R.N., and ALEXANDER D. CAMPBELL, B.A. . . . 208

ART. IX.—1. The London Medical Directory, 1845. Containing the name, address, qualification, official appointments, honorary distinctions, and literary productions of every Physician, Surgeon, and General Practitioner resident in London . . . . . . . . ib.

2. The Medical Directory of Great Britain and Ireland for 1845. . ib.

---

### PART THIRD.—Original Reports and Memoirs.

1. Report on the Progress of Pathology, Practical Medicine, and Therapeutics, during the years 1842-3-4. By JAMES RISDON BENNETT, M.D. Assistant Physician and Lecturer on Materia Medica and Therapeutics at St. Thomas's Hospital . . . . . . . . 209

2. Inflammation as it occurs in the different Tissues of the Eye; being a complement to the Author's Reports on Inflammation and the Healing Process. By T. WHARTON JONES, F.R.S. Lecturer on Anatomy, Physiology, and Pathology, at the Charing Cross Hospital; Corresponding Member of the Imperial and Royal Society of Physicians and Surgeons of Vienna, &c. . 273

---

Dr. Elliotson and Mesmeric Diagnosis . . . . . 287
Books received for review. . . . . . . 288

